# What Drives Customer Satisfaction, Loyalty, and Happiness in Fast-Food Restaurants in China? Perceived Price, Service Quality, Food Quality, Physical Environment Quality, and the Moderating Role of Gender

**DOI:** 10.3390/foods9040460

**Published:** 2020-04-08

**Authors:** Yongping Zhong, Hee Cheol Moon

**Affiliations:** Department of International Trade, Chungnam National University, 99 Daehak-ro, Yuseong-gu, Daejeon 34134, Korea

**Keywords:** food consumption, food service industry, satisfaction, loyalty, happiness, perceived price, service quality, food quality, physical environment quality, moderating role of gender

## Abstract

The fast-food service industry has been growing rapidly across China over the last few decades. In accordance with the rising consumption level in the country, Chinese customers care increasingly about their food choices. The purpose of this study is to investigate the factors that can influence customer satisfaction, loyalty, and happiness, with a particular focus on the moderating role of gender. Data were collected through an online survey completed by customers who visited Western fast-food restaurants (KFC, McDonalds, etc.) in China. The structural equation model was applied to test 12 hypotheses. Results showed that perceived price, food, service, and physical environment quality positively affected customer satisfaction. Perceived price can significantly influence customers’ judgement of the quality dimensions of a restaurant. Moreover, customer satisfaction and happiness can lead to a sense of loyalty. Happiness functions as a mediator between satisfaction and loyalty. Nonetheless, our findings indicated that customers’ perceptions of food quality based on price and satisfaction levels based on service quality differ significantly between the genders, which demonstrated that gender moderation exists in food consumption. This study will contribute to a better understanding of managerial and theoretical perspectives, which will be beneficial for subsequent research.

## 1. Introduction

China, a huge emerging market with great potential, has been growing very fast since joining the WTO (World Trade Organization) in the early 2000s. Over the past few decades, the consumption level of the middle class has been increasing. Meanwhile, Western fast-food chains are expanding rapidly, and eating in a Western fast food restaurant has become a trend among the younger generations [1]. As a result, competition within the catering industry has become more and more fierce. Today, Western fast-food giants are facing challenges from local restaurants that have expanded dramatically across China in recent years. In addition, with the improvement in living conditions, Chinese customers care increasingly about what, how, and where they eat, so competition between Western companies and local companies is inevitable. In order to compete with local food restaurants and generate greater profit, Western fast-food companies must pay more attention to price, service quality, food quality, and physical environment. Of these four factors, price is the most critically influential. Price may reflect service quality and even change customers’ purchasing behavior [2]. It can influence customers’ perception of restaurant quality [3]. These quality dimensions (service, food, and physical environment) of a restaurant are crucial determinants of customer satisfaction [4]. Maintaining customer satisfaction is very important because it can lead to repeat customers and increased sales [5,6]. A great amount of research in the service marketing field has focused solely on customer satisfaction and loyalty. However, there have been insufficient comprehensive studies conducted to establish how happiness and life satisfaction are related to consumer buying patterns [7]. However, happiness is assumed to constitute a higher level of customer satisfaction [8] and can improve people’s quality of life [9]. The concept of happiness has recently aroused increased attention from scientists working in various fields. Through a good dining experience, customers may improve their life quality and increase their happiness. A delightful dining experience can entice customers to revisit a restaurant. Investigating the role of happiness is significantly necessary for the food service industry.

According to international studies of addictive shopping, female customers contribute 70% of product sales [10], while in China, based on 2018 World Bank data [11], females accounted for 48.7% of the total population. Recently, female purchasing power has been increasing. Understanding gender differences is crucial for a country’s economic and social policy and strategies from a macro perspective, and it is also important in terms of company success from a micro perspective [12]. Individual customer characteristics can be used as marketing segments which allow companies to adjust and maintain specific strategies based on customer needs. Such demographic features can provide companies with more information for market segmentation to achieve better market penetration, while gender is always one of the most common marketing segments [13]. Gender differences have commonly been studied in psychological contexts, but how gender can affect customer perceptions and attitudes toward a restaurant is relatively less developed in service-marketing. It appears that studies of gender differences related to food consumption and dining experience have been very limited, but ignoring gender differences in food consumption may cause management problems.

Examining how perceived price, service quality, food quality, and physical environment quality can influence customer behavior, testing the implications of perceived price on restaurant quality dimensions, and studying the relationships in the proposed model will contribute to understanding the food service industry in China. This study intends to narrow down theoretical and practical gaps by developing an integrated model with a special focus on gender effects. If Western fast-food companies can understand customer behavior better, set more specific market segmentation, and launch marketing strategies targeting different genders, they are more likely to keep customers satisfied, happy, and loyal. This study consists of five parts: introduction, literature review and hypotheses, methodology, results, and discussions and conclusion.

## 2. Literature Review and Hypotheses

### 2.1. The Effect of Perceived Price, Food, Service, and Physical Environment Quality on Customer Satisfaction

Price refers to the amount of money that customers spend on a product or service. Generally speaking, price is the value that customers give up as an exchange for the benefits of using a product or service [14]. Price plays an important role in generating consumer satisfaction, as customers always evaluate the value of a service by its price [15]. Campbell (1999) [16] indicated that price fairness significantly impacts brand image; as a consequence, perceived price unfairness may cause negative behaviors, such as negative word of mouth and switching brands. Rothenberger (2015) [17] also suggested that customers’ negative perception toward unfair prices can cause dissatisfaction, decreased repurchasing behavior, negative word of mouth, and complaints.

Food quality is very significant in determining customer satisfaction and loyalty. Generally, food quality refers to several aspects including food presentation, taste, menu diversity, healthiness, and freshness [18]. A high level of food quality is a key marketing strategy which can satisfy and retain customers, and provide a happy purchasing experience for them. Food quality can have a considerable effect on customer satisfaction and behavioral intentions [19]. Several studies indicated that food quality can positively influence customer satisfaction [20,21,22].

Customers’ decisions and purchasing behaviors are closely related to their evaluation of the overall experience of a service or product [23]. Service quality can significantly affect customer satisfaction and loyalty, which is critical to a company’s success. High levels of service quality may lead to high customer satisfaction [24], but if the service performance fails to match customers’ expectations, dissatisfaction will occur [25]. 

Physical environment of a hotel or restaurant can strengthen the brand image of a company, reshape customers’ perceptions, and directly influence customer satisfaction [26]. According to Hanaysha (2016) [18], all tangible and intangible elements inside and outside of the restaurant are included in the concept of physical environment, including temperature, lighting, scent, noise, atmosphere, and music. He also suggested that a well-maintained physical environment can serve to maintain a restaurant’s existing customer base as well as attract new customers.

Customers’ perception of product, atmospherics, and service are closely related to their emotions (both positive and negative), and behavioral intentions based on the consumption experience in the restaurants [22,27]. Ambient elements (such as sound, smell, taste, touch), design elements (such as store decoration and layout), and social elements (such as interaction with member staff) can extensively impact customers behavior [22]. Lim (2010) [28] also indicated that a high food and service quality, along with a comfortable atmosphere, are very important to a restaurant because it may contribute to a higher satisfaction level and even influence customers’ subsequent behavior in the food service industry.

Based on the literature presented above, the following hypotheses are proposed:

**Hypothesis 1** **(H1).** *Perceived price has positive effects on customer satisfaction.*

**Hypothesis 2** **(H2).** *Food quality has positive effects on customer satisfaction.*

**Hypothesis 3** **(H3).** *Service quality has positive effects on customer satisfaction.*

**Hypothesis 4** **(H4).** *Physical environment has positive effects on customer satisfaction.*

### 2.2. The Effect of Perceived Price on Food, Service, and Physical Environment Quality

Price is the value that customers sacrifice to obtain a product or service [14,29]. Price includes information for evaluating the level of service which may influence customers’ purchasing behavior [2]. According to Ryu and Han (2010) [4], perceived price moderates the correlations between quality dimensions (food quality, service quality, and physical environment quality) and satisfaction, which means if the perceived price is reasonable, this may increase the customer satisfaction level regarding food, service, and physical environment quality. Price can influence customers’ value expectations of a restaurant [30]. Customers are not only affected by the actual price of a product or service stated on the price tag, but also affected by their own perceptions which are shaped in a comparative and subjective way [31]. The actual price will not increase the quality of a product or service, but it definitely will influence the subjective value [32]. When evaluating the quality of a product or service, the higher the price, the higher the quality the customers expect, because higher prices add equal value to the quality [33]. Even though the price perceived by the customers will effectively change customers’ expectations of food, service, and physical environment quality from a restaurant, little empirical research has tested how perceived price can influence customers’ judgement of restaurant quality directly, and the internal relationships between perceived price and quality dimensions remain unclear. Therefore, in this study, instead of investigating whether price moderates the relationships between these three quality dimensions and customer satisfaction, as Ryu and Han (2010) [4] sought to do, we intend to study how perceived price can directly and positively affect these quality dimensions. And the following hypotheses are proposed:

**Hypothesis 5** **(H5).** *Perceived price has positive effects on food quality.*

**Hypothesis 6** **(H6).** *Perceived price has positive effects on service quality.*

**Hypothesis 7** **(H7).** *Perceived price has positive effects on physical environment quality.*

### 2.3. Customer Satisfaction, Loyalty, and Happiness

Customer satisfaction can be defined as an overall assessment of a product or service based on the experience of purchasing and consuming it over time [34]. Service and product quality, pricing strategy, and store characteristics are the main factors that can affect customer satisfaction. Companies can achieve customer satisfaction and loyalty by providing good-quality products and services [6]. Satisfied customers tend to repurchase products and become loyal customers, and they are positively engaged in giving recommendations to other customers and less sensitive to price [34]. Moreover, once customers are satisfied with a product or brand, they are more likely to recommend the brand to others, and are more likely to repeatedly purchase that product instead of switching to other alternative brands [35].

Dick and Basu (1994) [36] noted that customer loyalty can be categorized in three ways: service loyalty, brand loyalty, and store loyalty. Other scholars, such as Bowen and Chen (2001) [37], stated that behavior (consistent, repetitive purchasing action), attitude (emotional and psychological connections), composite (a mixture of the two measures above, loyalty measured by customer preferences, repurchasing, word of mouth, and inclination to switch to other brands) are the three key elements in defining loyalty. Customers’ experience can be connected not only with functional dimension, but also other dimensions, such as sensorial, emotional, cognitive, behavioral, and relational aspects [22]. Repurchasing intentions are critically influenced by product satisfaction, and if satisfaction level increases, customer retention is a greater possibility [38]. In other words, satisfaction will lead to loyalty, and customer loyalty is a derivative of customer satisfaction [34].

During food consumption, an exceptional dining experience will not only make customers satisfied but also happy. Happiness is a positive judgment from the subjective perspective of an individual who feels satisfied with his current situation, and consumer happiness refers to the emotions of consumers which are related to consumption activities [39]. Additionally, customer happiness is defined as conceptions of customers based on the extent to which their well-being and quality of life quality are improved [9]. The purpose of service marketing is shifted from making customers satisfied to increasing their happiness, which reaches beyond the concept of satisfaction [40]. Happiness is considered as a higher level of customer satisfaction [8]. Satisfaction accompanied by concrete activities that relate to life domains can enhance customer happiness [41]. For example, consumers engaged in materialistic consumption implicitly believe that this kind of consumption may increase their self-esteem [42]. Nicolao, Irwin, and Goodman (2009) [43] suggested that many experiential purchases involve activities with other people including friends and family, and positive social interaction is a major source of happiness. In other words, the purchasing experience can bring customers happiness, and in order to achieve greater happiness, customers may repeatedly engage in consumption activities. Happiness could lead satisfied customers to be more loyal, but none of the previous studies have tested if happiness plays a mediating role between satisfaction and loyalty, so the relationship between these factors remains uncertain. In this study, in order to understand the connections between these three variables more deeply, we will test the mediation effect of happiness.

Therefore, based on preceding studies, the following hypotheses are proposed:

**Hypothesis 8** **(H8).** *Customer satisfaction has positive effects on customer loyalty.*

**Hypothesis 9** **(H9).** *Customer satisfaction has positive effects on happiness.*

**Hypothesis 10** **(H10).** *Happiness has positive effects on customer loyalty.*

**Hypothesis 11** **(H11).** *Happiness mediates the relationship between satisfaction, loyalty, and happiness.*

### 2.4. Gender Differences

Gender is considered to be critically influential in the purchasing process. Gender as a marketing segment is a decisive factor in terms of market penetration. As more and more women are becoming more powerful and active in purchasing activities, ignoring gender differences when devising marketing strategies may bring problems. In some studies, women tend to be relatively more emotional [44], while men are considered to be relatively more aggressive and autonomous [45]. Argyle and Henderson (1984) [46] pointed out that female customers generally tend to give higher ratings for performance than males. In addition, females may intend to have affiliation with people and place more emphasis on social interactions when they are served by others [47]. Furthermore, gender differences can lead to different food choices. Some studies have shown that unlike most males, females pay more attention to food and they are more concerned about food choices [48,49]. Gender differences are closely related to how male and female customers will evaluate a restaurant’s quality, and can have implications for their subsequent behavior. Studying the moderating effects of gender on customers’ dining experience and food consumption is very necessary as it may contribute to a better understanding of different gender groups, which will also minimize research gaps within prior studies and reduce market segmentation problems.

#### 2.4.1. Perceived Price, Restaurant Quality Dimensions, and Gender

Referring to previous literature, evidence of how gender influences peoples’ conception of fairness toward perceived price seems to be complicated. Some scholars, for example, Snipes, Thomson, and Oswald (2006) [50], indicate that male customers are inclined to assign higher scores for fairness. Additionally, according to Beldona and Namasivayam (2006) [51], who studied demand-based pricing and gender differences in perceived fairness, females tend to rate lower for perceived fairness, which means that they are more likely to perceive a price as unfair. Others argued differently; for example, Adams (1965) [52] claimed that females are more likely to be sensitive when it comes to fairness under similar contexts, while Lee and Farh (1999) [53] pointed out that there are not any gender differences in justice-outcome relationships and any actual differences observed may result from contextual and study design factors. However, previous studies presented contradictory arguments, and most of them mainly focused on the differences in judgements of fairness according to gender, but insufficient studies have investigated how gender differences may moderate the relationship between perceived price and quality dimensions of a restaurant. Thus, it remains unclear whether gender is a factor that moderates the correlations between perceived price and customers’ judgements of the attributes of a restaurant.

Some prior studies mentioned gender differences in evaluating product quality and physical environment quality, and suggested that males and females vary in terms of fashion consumption, with female customers being more sensitive to product quality and physical elements of product attributes [54]. Gender differences also play an important role in food consumption. Some studies have indicated that female and male customers generally have different requirements for food, suggesting that females place more emphasis on attributes of food quality, such as taste, presentation, and menu variety, but males place more emphasis on portions [55,56]. Buda, Sengupta, and Elkhouly (2006) [57] noted that gender and education have implications for service quality dimensions that include tangible elements, such as physical facilities and equipment. Holbrook (1986) [58] also indicated that females are more sensitive to visual and romantic elements than males. Still, studies related to how gender can influence customers’ judgement of food quality and physical environment of a restaurant remain comparatively limited in quantity.

The literature related to gender effects on service quality tends also to be very complicated. Some studies have found that female customers tend to give lower ratings for service quality than males [50,59,60]. However, some other scholars have differing views toward service quality based on gender. For example, Peter and Olson’s (1999) study [61] suggested that female customers can be more sensitive than males with regard to relational aspects of a service. Moreover, Ndhlovu and Senguder’s (2002) study [62] found that male and female customers’ perceptions do not significantly differ regarding hotel staff service.

#### 2.4.2. Satisfaction, Loyalty, Happiness, and Gender

Based on the American Customer Satisfaction Survey Index, female customers’ satisfaction level is generally higher than that of males [63], which can be explained by the fact that females tend to have more communal concerns and are more in need of connections and harmonious relationships with other people [64]. Additionally, men have a higher tendency than women to take risks, and are expected to be involved socially in risky behavior. Male tend to be less worried about switching brands and trying new things, so they may be less likely to become loyal to a provider [65], but compared to males, females are more likely to revisit a restaurant if they are satisfied [66].

Some psychological studies have indicated that gender does not influence people’s perception of happiness. According to Chui and Wong (2016) [67], female happiness and life satisfaction are related to family and other social ties, but male happiness may be more closely connected with feelings (self-concept). They investigated the effects of gender on happiness and life satisfaction in Hong Kong, but found that gender is relatively uninfluential vis-a-vis happiness. Happiness has been researched within other social science fields for a long time, but in the service-marketing field, no study has investigated whether gender plays a significant role in shaping customers behaviors toward happiness.

Therefore, in order to fill this research gap and investigate if gender moderates the relationship between these variables (perceived price, service quality, food quality, physical environment quality, customer satisfaction, loyalty, and happiness), we would like to propose the following hypothesis:

**Hypothesis 12** **(H12).** *Gender moderates the relationships between perceived price, service quality, food quality, physical environment quality, customer satisfaction, loyalty, and happiness.*

## 3. Methodology

### 3.1. Questionnaire

The questionnaire was divided into 7 parts, each part investigates one market factor: perceived price (PP), food quality (FQ), service quality (SQ), physical environment quality (PQ), customer satisfaction (SA), customer loyalty (LY), and happiness (HA). A 5-point Likert-type scale that ranged from 1 (strongly disagree) to 5 (strongly agree) was adopted in this study and which was used previously by Kasiri et al. (2017) [68] to measure service quality, customer satisfaction, and loyalty. Hanaysha (2016) [18] also used s 5-point Likert-type scale to measure food quality, price fairness, physical environment, and satisfaction. Twenty-three items (see Table 1) were used to measure the aforementioned 7 factors. Most of the items were adapted from previous studies, but considering the limited literature relating to happiness, this variable was measured using two items from Gong and Yi (2018) [9] and a self-developed item.

### 3.2. Data Collection

Data were mainly collected from Western fast-food restaurants (KFC, McDonalds, etc.) in China. Before beginning research, the questionnaire was translated into Chinese by the authors in order to make sure that participants fully understood the research content. The questionnaires were sent to potential participants through a Chinese survey website, which is operated by Tencent company, aiming to act as a social platform for online survey. The data were randomly collected online during September of 2019. In total, 325 volunteers participated in this study. However, as fast-food is more popular among the young generations and they have more access to the internet, the majority of the respondents were aged 21–40. Most of the participants took about 10 minutes to complete the survey, but only 305 of them completed the questionnaires properly. Questionnaires that contained incomplete or inappropriate answers were excluded from analysis.

The descriptive data (see Table 2) show that 41.3% of the participants were male and 58.7% were female. Approximately 63.0% of respondents were from the age group 21–30 years old, followed by 20.3% aged 31–40, 13.1% aged 20 and under, 3.3% aged 41–50, and only 0.3% of the respondents were above 50. The educational backgrounds of respondents were quite varied; most of them had undergraduate degrees (50.8%) or graduate degrees and above (22%). Of these participants, 20.6% of them visited fast-food restaurants 1–2 times every 6 months and 13.1% visited 2 times every 3 months. Most respondents visited fast-food restaurants quite often, as 19% of them would do so once per month, 26.2% 2–3 times monthly, 16.1% 1–3 times weekly, and 4.9% more than 3 times weekly. The monthly income of the majority was more than 3000 RMB; 24.3% of them earned 5001–8000 RMB per month, followed by 3001–5000 RMB (23.6%) and more than 8000 RMB (15.4%).

## 4. Analysis and Results

In order to test the proposed hypotheses, this study adopted a PLS-SEM (Partial least squares–structural equation modeling) approach [71,72,73,74]. The PLS-SEM method has been used widely in management research in recent years [75,76,77,78]. PLS has minimal restrictions for sample size and residual distributions [79]. In general, PLS is suitable for explaining complicated relationships because it can avoid inadmissible solutions and factor indeterminacy [80]. In this study, because 7 variables were included to study complicated relationships between these variables and moderating effects, the PLS-SEM method was deemed to be relatively suitable and beneficial for the research purpose. The study utilized structural equation modeling (SEM) using SmartPLS3.2.8 software. Partial least squares (PLS) were used with a 5000-subsample bootstrapping procedure, which was suggested by Hair et al. (2016) [81]. In addition, SPSS 24 was used during data analysis for exploratory factor analysis and descriptive analysis.

### 4.1. Exploratory Factor Analysis

The exploratory factor analysis used SPSS 24. In this study, exploratory factor analysis is conducted to reduce data to a smaller set of summary variables and to explore the potential theoretical structure of the phenomena, which can also examine the relationship between different variables. Based on Kaiser-Meyer-Olkin (KMO) and Bartlett’s test results (Table 3a), the sampling adequacy was 0.944 and the significant level was below 0.001, indicating the data were suitable for exploratory factor analysis. Principal component analysis (extraction method) and Equamax with Kaiser normalization (rotation method) were used to extract factors, which were suggested by Maroco, A. L. and Maroco, J. to investigate customer satisfaction and loyalty [82]. Equamax is a combination of the varimax method and the quartimax method, which can simplify the factors and the variables. This method can minimize the number of variables that load heavily on an item and the number of items needed for explaining a variable. Based on this method, factors, LY3, SA3, PQ1 were excluded from the next-step analysis after the exploratory factor analysis due to cross-loading problems (see Table 3b). The remaining 23 items were applied in further analysis as follows.

### 4.2. SEM Model Analysis

The fitness of the model was evaluated by the root mean square residual (SRMR) and normed fit index (NFI). If SRMR is less than 0.08, it indicates a reasonable fit [83]. The SRMR of this model was 0.052, which indicates good model fitness. Bentler and Bonett (1980) [84] suggested that the NFI value should range from 0 to 1, but the closer the NFI is to 1, the better the fitness is. As the NFI was 0.826 in this model, it represented reasonable model fitness.

Cronbach’s Alpha was included to assess the reliability of each construct. The overall Cronbach’s Alpha values for each construct was above 0.7, which means that Cronbach’s Alpha values were within the acceptable level [85] and indicate a high level of internal consistency of each variable (see Table 4). If the rho A value is more than 0.7, it means a regular fit [86]; thus, rho_A value of this model was within the acceptable level. The outer factor loadings were above 0.5 (see Table 4), which were consistent with the recommended level [87] and showed good convergent validity. In order to test the PLS-SEM models, AVE (average variance extracted) and CR (construct reliability) were evaluated. In the measurement model, the recommended AVE level was above 0.5 and the CR level was above 0.7, which matches Bagozzi and Yi’s (1988) [88] suggestion indicating good construct reliability (see Table 4). The discriminant validity was evaluated in adherence with Fornell and Larcker’s (1981) [89] theories where AVE’s square root of each construct should exceed the correlation value and results showed good discriminant validity (see Table 5).

### 4.3. Hypotheses Test Results

**Figure 1:**
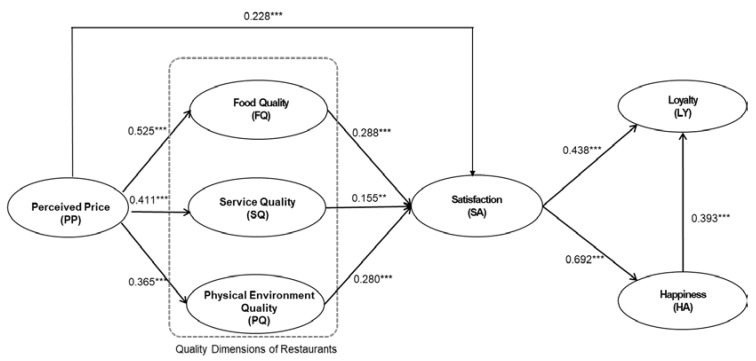
PLS-SEM (Partial least squares - structural equation modeling) whole group results (note: ** *p* < 0.01; *** *p* < 0.001).

As shown in Table 6, 10 hypotheses were statistically significant. Perceived price (β = 0.228, *p* < 0.001), food quality (β = 0.288, *p* < 0.001), service quality (β = 0.155, *p* < 0.01), and physical environment quality (β = 0.280, *p* < 0.001) were positively related to customer satisfaction, supporting H1, H2, H3, and H4. The findings for H5, H6, and H7 demonstrated that perceived price had positive effects on food quality (β = 0.525, *p* < 0.001), service quality (β = 0.411, *p* < 0.001), and physical environment (β = 0.365, *p* < 0.001), which supported H5, H6, and H7. The data also indicated that customer satisfaction could positively influence customer loyalty (β = 0.438, *p* < 0.001) and happiness (β = 0.692, *p* < 0.001); therefore, H8 and H9 was supported. Furthermore, the coefficient between happiness and customer loyalty was 0.393 (*p* < 0.001), which indicated that happiness could positively impact loyalty, so H10 was supported (Figure 1).

### 4.4. Moderating Effects of Gender

**Figure 2:**
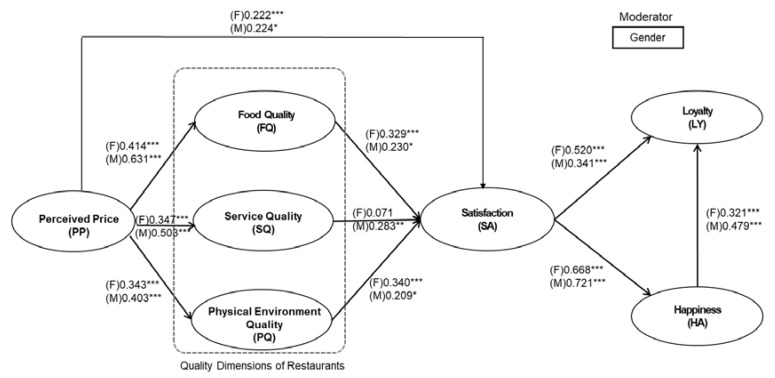
PLS-SEM results for female and male customers (note: *p < 0.1; **p < 0.01; ***p < 0.001; F = female; M = male).

Multigroup analysis with parametric testing was performed in PLS to test the gender moderating effects of 10 paths (see Table 7, Figure 2). Two out of ten paths (H3 and H5) proved to differ significantly between two groups (*p* < 0.1). Gender moderated the relationship between service quality and satisfaction with male customers (β^f^ = 0.071, β^m^ = 0.283, *p* < 0.1), due to service quality having a stronger influence on male customers than female customers. Gender also had moderating effects on the relationship between perceived price and food quality (β^f^ = 0.414, β^m^ = 0.631, *p* < 0.05). In terms of evaluating food quality, female customers tended to be less affected by price than males.

Statistically gender did not moderate the relationship between perceived price and other factors for path H1, H6, and H7, but male customers, with slightly higher coefficients on the paths, were more likely to be influenced by perceived price during the evaluation of satisfaction level (β^f^ = 0.222, β^m^ = 0.224), service quality (β^f^ = 0.347, β^m^ = 0.503), and physical environment quality (β^f^ = 0.343, β^m^ = 0.403). Meanwhile, gender moderating effects were not significant for paths H2 and H4, but female customers’ satisfaction level was influenced to a slightly greater extent by food quality (β^f^ = 0.329, β^m^ = 0.230) and physical environment quality (β^f^ = 0.340, β^m^ = 0.209). Finally, gender did not moderate the relationship between satisfaction, loyalty, and happiness (H8, H9, and H10), but the path coefficient of H8 indicates that female customers were more likely to become loyal customers if satisfied, with a slightly stronger relationship between satisfaction and loyalty (β^f^ = 0.520, β^m^ = 0.341). Even though the path coefficients of H9 and H10 were not significantly different, the data suggested that if male customers were satisfied, they would have a slightly higher probability of becoming happy (β^f^ = 0.668, β^m^ = 0.721); consequently, if they were happy, they would be more inclined to be loyal than females (β^f^ = 0.321, β^m^ = 0.479).

### 4.5. Happiness Mediation Test

In order to investigate the mediating role of happiness, we conducted a mediating test to see if happiness could act as a mediator between satisfaction and loyalty. As shown in Table 8 (bootstrapping data from PLS), the indirect effects (*p* < 0.001), direct effects (*p* < 0.001), and total effects (*p* < 0.001) were significant for the whole group, which indicates that happiness mediated the relationship between satisfaction and loyalty, so H11 was supported.

## 5. Discussions and Conclusions

### 5.1. Discussions and Theoretical Implications

The purpose of this study was to investigate how the determinants (perceived price, service quality, food quality, physical environment quality) could influence customer satisfaction, loyalty, and happiness with a special focus on moderating effects of gender. One of the important findings was that quality dimensions of a restaurant and perceived price had positive effects on customer satisfaction. These findings are consistent with Hanaysha (2016) [18], and Qin and Prybutok’s (2009) [21] research into fast-food restaurants.

Moreover, we found that a reasonable price could positively and directly affect customers’ perceptions toward quality of a restaurant. The result is partly consistent with Ryu and Han’s (2010) [4] research, in which they argued that perceived price plays a moderating role in acting between customers’ satisfaction and quality dimensions (food, service, and physical environment quality). However, in this study, we proposed a new model and verified that perceived price as an independent variable (not a moderator) could not only directly affect customer satisfaction, but also the quality dimensions of a restaurant. With regards to previous studies, there has been insufficient empirical research testing how strongly perceived price can impact customers’ perceptions of restaurant quality dimensions, and their internal relationships remains unclear. This study may contribute to a better understanding of the implications of perceived price in the food service industry and fill research gaps accordingly.

The results also showed that satisfaction could have positive effects on loyalty. This was consistent with several previous studies [4,5,6]. In addition, the results revealed that satisfaction was positively related to customer happiness, which is similar to Gong and Yi’s (2018) [9] findings in their recent research. However, they also suggested that loyalty could positively affect happiness, but in this study, we found that, conversely, happiness could positively influence loyalty. This may be explained by the fact that being involved in purchase activities can bring happiness to customers [42,43], a happy shopping experience can lead to repurchasing. Furthermore, we found that happiness could function as a mediator between satisfaction and loyalty. This new finding confirmed that satisfied customers may become loyal when they are happy with the dining experience. Happiness, an essential factor in increasing customers’ quality of life, reaches beyond the concept of satisfaction. Compared to the definitions of satisfaction that are relatively well established by scholars, there have been very few studies related to happiness, and the current literature still exhibits some inconsistencies in defining customer happiness [90]. As most of the literature related to happiness comes from other social science fields, such as psychology, the effects of happiness in the marketing field remained undeveloped. Investigating the role of happiness is likely to contribute to a better understanding of the concept within marketing research.

Additionally, the results revealed the moderating role of gender, which can moderate the relationship between perceived price and food quality, with males displaying a stronger relationship than females. Most previous studies only focused on gender difference in judgements of fairness [50,51,52,53], and no study examined how gender difference can influence customers’ evaluations of food quality based on perceived price. In this study, however, we found that females tended to be less influenced by price when evaluating the food quality of a restaurant. Moreover, the results revealed that gender can also moderate the relationship between service quality and satisfaction, with a stronger relationship existing among males. This result opposes the findings of Ma, QU, and Eliwa (2014) [6] who found that services had a stronger effect on satisfaction among female customers than male customers in American fine-dining restaurants. However, under different contexts, the moderating effects of gender may vary between countries, it may explain why the effects of service quality on satisfaction were not so strong comparatively among female customers in our findings. There have been insufficient studies investigating gender differences in food consumption and dining experience. Therefore, this study’s research into how gender can affect customers’ evaluations of a restaurant’s quality dimensions and how gender can affect customers’ emotions and behaviors may fulfil such theoretical needs.

### 5.2. Managerial Implications

For these fast-food franchise giants, they might need to make efforts to improve restaurant quality dimensions (food, service, and physical environment quality), since these are very influential to customers’ behavior. Managers should place more emphasis on the freshness, taste, and presentation of the food. Staff members should be well trained to be professional. It is also very important to keep the restaurant clean and comfortable. However, setting an appropriate price strategy is of critical importance, as we found that perceived price will not only directly affect customers’ satisfaction level but also their expectations of food, service, and physical environment quality. Thus, price should be reasonable compared to the quality. Moreover, for managers, it should be acknowledged that happiness is something that extends beyond notions of satisfaction and can moderate the relationship between satisfaction and loyalty. Satisfied and happy customers have strong propensities to becomes loyal and be more willing to revisit the restaurant and recommend it to other customers. It is very necessary for managers to continuously adopt various strategies to meet customer needs, and let customers not only to feel satisfied but also happy in order to encourage loyalty. Consequently, by maintaining these loyal customers, companies that struggled for customer retention could generate greater profit.

Considering gender differences, sometimes marketers should regard females and males as different segmentations, meaning that they may need to adjust their marketing strategies based on gender. Compared to female customers, males are more likely to be influenced by price when they evaluate food quality, so for products targeting male customers, price fairness should be considered more carefully than for other products. In addition, as male customers are more sensitive to service quality, and their satisfaction level is more likely to be affected by service quality, it is important to understand the needs of male customers in detail and focus more on the service attributes that male customers care about the most, especially in the service industry. Both female and male customers are influential decision-makers in food consumption. Therefore, their needs are equally important. Awareness of gender differences can contribute to a better understanding of female and male customer needs and behaviors, which may lead to improved market segmentation and increased market shares.

### 5.3. Limitations and Future Research

This study has a few limitations. Firstly, for purposes of sampling process convenience, the majority of participants were young people. It would have been preferable if samples represented more varied age groups. Secondly, this study only focused on Western fast-food restaurants in China, and as such, some of the findings from this study may not accord with those derived from studies of other countries or other types of restaurants. Thirdly, only four antecedents (price, food, service, and physical environment quality) of satisfaction were investigated in this study, but there may be more variables that could be included based on other literature. Fourth, we investigated the moderating role of gender and the mediating role of happiness, but other moderators or mediators may exist in addition to these.

We would like to suggest that future studies should gather surveys from a larger scale in order to include more participants of differing age and background. Moreover, considering the diversity of countries and restaurant types, future research could be conducted not only in the fast-food industry but across a wider range of restaurants and countries. This study only tested four antecedents, but there may be more factors, such as location or delivery service, which could act as antecedents of customer behaviors. Last but not least, besides the moderating effects of gender, alternatively, country or age may moderate the proposed relationships. In addition, other mediators between customer satisfaction and loyalty, such as brand image and perceived switching cost, could be further examined. Future studies may include more variables in order to extend this model and gain further insight.

## Figures and Tables

**Table 1:** Questionnaire and sources.

Variable	Observed Variables	Items	Source
Perceived Price (PP)	PP1	The price of the fast food is reasonable.	Adapted from [18,69,70]
	PP2	Based on the food, the price here is fair.	
	PP3	The price of the fast food is affordable.	
Food Quality (FQ)	FQ1	The food smells good.	Adapted from [5,18]
	FQ2	The food is delicious.	
	FQ3	The food is fresh.	
	FQ4	The food looks attractive to me.	
Service Quality (SQ)	SQ1	Staff members are friendly.	Adapted from [5,21]
	SQ2	Staff members are very helpful.	
	SQ3	Staff members serve quickly and promptly.	
	SQ4	I feel comfortable with staff members’ service.	
Physical Environment Quality (PQ)	PQ2	The restaurant environment is clean.	Adapted from [5,18]
	PQ3	The lighting in the restaurant is comfortable.	
	PQ4	The temperature in the restaurant is comfortable.	
Satisfaction (SA)	SA1	The overall experience of this fast-food restaurant is satisfying.	Adapted from [5,18]
	SA2	I think my decision to visit this restaurant was a wise one.	
	SA3	This restaurant meets most of my expectations.	
Loyalty (LY)	LY1	I will continue to visit this restaurant.	Adapted from [5,9]
	LY2	I will recommend this restaurant to others.	
	LY3	I will say positive things about this restaurant to others.	
Happiness (HA)	HA1	I think visiting this fast-food restaurant will contribute to customer happiness.	Adapted from [9]
	HA2	By visiting this restaurant, customers’ quality of life will be improved.	
	HA3	This restaurant provides a happy and enjoyable dining experience for my family and friends.	Self-developed

**Table 2:** Sample profile.

Variable		Frequency	Percent %
Gender	Male	126	41.3
	Female	179	58.7
Age	20 and under	40	13.1
	21–30	192	63.0
	31–40	62	20.3
	41–50	10	3.3
	above 50	1	0.3
Education	below high school	13	4.3
	high school/vocational school/technical school	31	10.2
	junior college	39	12.8
	undergraduate	155	50.8
	graduate and above	67	22.0
Frequency of Visiting Fast-Food Restaurants	1–2 times every 6 months	63	20.6
	2 times every 3 months	40	13.1
	once a month	58	19.0
	2–3 times a month	80	26.2
	1–3 times a week	49	16.1
	more than 3 times a week	15	4.9
Monthly Income	less or equal to 1000 RMB	29	9.5
	1001–1500 RMB	32	10.5
	1501–2000 RMB	30	9.8
	2001–3000 RMB	21	6.9
	3001–5000 RMB	72	23.6
	5001–8000 RMB	74	24.3
	more than 8000 RMB	47	15.4
	total	305	100.0

**Table:** (**a**)

KMO and Bartlett’s Test			
KMO Measure of Sampling Adequacy	Bartlett’s Test of Sphericity		
0.944	Approx. Chi-Square	Df	Sig.
	4095.278	253	0.000

**Table:** (**b**)

Rotated Component Matrix ^a^							
	Component						
	1	2	3	4	5	6	7
SQ1	0.782						
SQ3	0.757						
SQ2	0.683						
SQ4	0.682						
PP1		0.828					
PP2		0.816					
PP3		0.788					
FQ1			0.761				
FQ4			0.707				
FQ2			0.682				
FQ3			0.546				
HA2				0.782			
HA3				0.666			
HA1				0.657			
PQ3					0.757		
PQ4					0.741		
PQ2					0.721		
LY1						0.752	
LY4						0.630	
LY2						0.610	
SA4							0.663
SA2							0.661
SA1							0.639
Extraction Method: Principal Component Analysis. Rotation Method: Equamax with Kaiser Normalization.							

Note: Df: degrees of freedom; Sig.: Significance. Factor loadings below 0.5 were not shown. ^a^ Rotation converged in 15 iterations.

**Table 4:** Outer loading, construct reliability, and validity results.

Variables	Outer Loadings (CFA)		Cronbach’s Alpha	rho_A	Composite Reliability	Average Variance Extracted (AVE)
perceived price	PP1	0.868	0.852	0.853	0.91	0.771
	PP2	0.884				
	PP3	0.883				
food quality	FQ1	0.827	0.85	0.85	0.899	0.69
	FQ2	0.863				
	FQ3	0.799				
	FQ4	0.832				
service quality	SQ1	0.804	0.846	0.851	0.896	0.684
	SQ2	0.849				
	SQ3	0.821				
	SQ4	0.832				
physical environment quality	PQ2	0.844	0.791	0.793	0.878	0.706
	PQ3	0.861				
	PQ4	0.815				
satisfaction	SA1	0.859	0.822	0.824	0.894	0.738
	SA2	0.882				
	SA4	0.835				
loyalty	LY1	0.835	0.825	0.826	0.895	0.741
	LY2	0.884				
	LY4	0.862				
happiness	HA1	0.876	0.854	0.855	0.911	0.774
	HA2	0.880				
	HA3	0.885				

**Table 5:** Discriminant validity.

	FQ	HA	LY	PP	PQ	SA	SQ
food quality	0.831						
happiness	0.615	0.88					
loyalty	0.591	0.696	0.861				
perceived price	0.525	0.513	0.458	0.878			
physical environment quality	0.594	0.533	0.539	0.365	0.84		
satisfaction	0.669	0.692	0.71	0.546	0.613	0.859	
service quality	0.609	0.51	0.544	0.411	0.506	0.566	0.827

**Table 6:** Hypotheses test results.

	Hypotheses	β	STDEV	T Statistics	*p* Values	Result
H1	perceived price → satisfaction	0.228	0.054	4.255	0.000	accepted
H2	food quality → satisfaction	0.288	0.068	4.253	0.000	accepted
H3	service quality → satisfaction	0.155	0.058	2.663	0.008	accepted
H4	physical environment quality → satisfaction	0.280	0.054	5.209	0.000	accepted
H5	perceived price → food quality	0.525	0.052	10.037	0.000	accepted
H6	perceived price → service quality	0.411	0.053	7.809	0.000	accepted
H7	perceived price → physical environment quality	0.365	0.046	7.942	0.000	accepted
H8	satisfaction → loyalty	0.438	0.061	7.178	0.000	accepted
H9	satisfaction → happiness	0.692	0.03	23.138	0.000	accepted
H10	happiness → loyalty	0.393	0.065	6.063	0.000	accepted

Note: STDEV: Standard Deviation

**Table 7:** PLS-SEM multigroup analysis results.

Hypotheses		Path Coefficients (β)		*p*-Values		STDEV		Path Coefficients-Diff
		(F)	(M)	(F)	(M)	(F)	(M)	*p*-Value
H1	perceived price → satisfaction	0.222	0.224	0.000	0.012	0.058	0.089	0.980
H2	food quality → satisfaction	0.329	0.230	0.000	0.036	0.083	0.111	0.465
H3	service quality → satisfaction	0.071	0.283	0.332	0.002	0.073	0.090	0.064 *
H4	physical environment quality → satisfaction	0.340	0.209	0.000	0.015	0.071	0.089	0.247
H5	perceived price → food quality	0.414	0.631	0.000	0.000	0.065	0.067	0.024 *
H6	perceived price → service quality	0.347	0.503	0.000	0.000	0.064	0.076	0.118
H7	perceived price → physical environment quality	0.343	0.403	0.000	0.000	0.064	0.061	0.512
H8	satisfaction → loyalty	0.520	0.341	0.000	0.000	0.085	0.079	0.139
H9	satisfaction → happiness	0.668	0.721	0.000	0.000	0.041	0.044	0.390
H10	happiness → loyalty	0.321	0.479	0.000	0.000	0.080	0.095	0.207

Note: * *p* < 0.1; STDEV: Standard Deviation; F = female, M = male.

**Table 8:** Mediating effect results.

Group	Stage		Effects		
	1st (SA→HA)	2nd (HA→LY)	Indirect Effects	Direct Effects	Total Effects
whole group	0.692 ***	0.393 ***	0.272 ***	0.438 ***	0.710 ***
female	0.668 ***	0.321 ***	0.215 ***	0.520 ***	0.735 ***
male	0.721 ***	0.479 ***	0.345 ***	0.341 ***	0.686 ***

Note: *** *p* < 0.001.
